# Mesenchymal stem cell therapy in bovine reproduction: Mechanistic insights, clinical applications, and translational challenges

**DOI:** 10.14202/vetworld.2025.2888-2899

**Published:** 2025-09-30

**Authors:** Teguh Ari Prabowo, Zultinur Muttaqin, Herdis Herdis, Santoso Santoso, Tri Puji Priyatno, Pradita Iustitia Sitaresmi, Tatan Kostaman, Mohammad Firdaus Hudaya, Santiananda Arta Asmarasari, Diana Andrianita Kusumaningrum, Nurul Azizah, Suyatno Suyatno

**Affiliations:** Research Center for Animal Husbandry, National Research and Innovation Agency, Soekarno Science and Technology Park, Cibinong, West Java 16911, Indonesia

**Keywords:** cattle reproduction, infertility, mesenchymal stem cells, ovarian function, regenerative therapy

## Abstract

Reproductive problems such as infertility, endometritis, and ovarian cysts are major causes of economic loss in cattle. Traditional treatments, including antibiotics and hormonal therapies, often show limited effectiveness and raise concerns about resistance and animal health. Mesenchymal stem cells (MSCs) offer a promising alternative because of their ability to regenerate tissues, modulate immune responses, and improve reproductive function. This review summarizes recent studies and consolidates emerging preclinical and clinical insights into MSC-based reproductive therapy in cattle. MSCs help repair ovarian and uterine tissues by migrating to damaged areas, reducing inflammation, releasing growth factors, and protecting against oxidative stress. Different methods of therapy, such as intrauterine infusion, intravenous delivery, and intraovarian injection, have been tested with encouraging results. For example, MSC therapy has improved pregnancy rates in cattle with endometritis and restored ovarian activity in animals with cystic ovaries. In addition to cell-based therapies, MSC-derived secretomes and exosomes demonstrate strong potential as cell-free approaches, offering regenerative effects with reduced risks. Despite these advances, challenges remain in translating MSC therapy to clinical practice. Variability in outcomes, technical expertise required for administration, and regulatory approval are major barriers. More studies are needed to standardize treatment protocols, evaluate long-term safety, and develop cost-effective strategies. Overall, MSC therapy represents a novel and sustainable approach for improving cattle fertility and herd productivity, offering an important step forward in veterinary reproductive biotechnology.

## INTRODUCTION

Reproductive health is essential for maintaining the productivity and profitability of the cattle industry. Disorders such as endometritis, cystic ovarian disease (COD), and infertility significantly impair reproductive efficiency and result in considerable economic losses, particularly in intensive production systems [1]. Conventional management strategies, including antibiotics and hormonal treatments, are widely employed to address these conditions. However, their effectiveness is often limited, with challenges such as antibiotic resistance, inconsistent outcomes, and potential adverse effects on animal health [2].

Over the past decade, stem cell-based approaches, especially those using mesenchymal stem cells (MSCs), have attracted increasing attention as innovative regenerative therapies [3]. Despite this growing interest, a comprehensive synthesis of their underlying mechanisms and practical applications in bovine reproduction remains scarce.

Stem cells possess the remarkable ability to differentiate into specialized cell types [4]. Among them, MSCs are among the most extensively studied, offering broad therapeutic potential due to their multipotent nature [5]. They contribute to tissue repair through several mechanisms: differentiation into functional cells, cell fusion, secretion of paracrine factors such as cytokines and growth factors and transfer of bioactive molecules through exosome-like vesicles [6]. Studies in multiple animal models, including mice, rabbits, pigs, and cattle, have demonstrated the mechanistic roles and therapeutic efficacy of MSCs [7].

In reproductive medicine, MSCs are particularly promising for repairing damaged ovarian and endometrial tissues [8]. Experimental evidence shows that they improve oocyte quality and support endometrial regeneration in preclinical models [9]. Mouse studies further suggest that MSC therapy enhances the expression of genes regulating angiogenesis and vascular development in the ovaries [10]. Post-transplantation analyses confirm MSC accumulation in the thecal and primordial follicle regions [11], while elevated estrogen (E2) and anti-Müllerian hormone levels in ovarian insufficiency models highlight their role in restoring ovarian function [12].

By combining regenerative and immunomodulatory properties, MSCs present a sustainable strategy for treating bovine reproductive disorders [13]. However, their translation into clinical practice is limited by challenges such as high production costs, lack of standardized administration protocols, and regulatory restrictions. Therefore, a comprehensive review is needed to consolidate current knowledge on MSC applications and regenerative mechanisms in cattle reproduction [14].

Although conventional treatments such as antibiotics and hormonal therapies are widely used to manage reproductive disorders in cattle, their effectiveness is limited by increasing antimicrobial resistance, inconsistent therapeutic outcomes, and possible adverse effects on animal health. In recent years, MSCs have gained attention as regenerative alternatives due to their multipotent differentiation capacity, immunomodulatory properties, and ability to secrete bioactive factors that promote tissue repair. Several experimental studies in animal models and cattle have shown encouraging results, including restoration of ovarian function, improved follicular development, and enhanced embryo production. However, despite this growing body of evidence, the application of MSCs in bovine reproductive medicine remains at an early stage. There is currently a lack of comprehensive synthesis that integrates mechanistic insights with clinical outcomes; evaluates the comparative advantages of different delivery methods (intrauterine, intravenous, intraovarian, and secretome-based); and critically discusses translational barriers such as cost, standardization, biosafety, and regulatory approval. Most available reports are scattered across experimental studies, and few have systematically addressed how MSCs can be advanced from preclinical promise to practical veterinary interventions. This fragmented knowledge base limits the development of standardized therapeutic protocols and hinders clinical adoption in cattle reproduction. Despite extensive experimental use of MSCs in reproductive biomedicine, a focused synthesis of their therapeutic applicability in cattle remains scarce. This review uniquely consolidates evidence from preclinical and clinical studies to elucidate the mechanistic and translational potential of MSCs in bovine reproductive health.

This review aims to consolidate current knowledge on the therapeutic potential of MSCs in bovine reproduction. Specifically, it seeks to (1) summarize the major reproductive disorders in cattle that may benefit from MSC-based therapy; (2) characterize the biological properties and mechanisms by which MSCs contribute to tissue regeneration, immunomodulation, and reproductive recovery; (3) evaluate the evidence supporting different therapeutic approaches, including direct cell transplantation and cell-free strategies using MSC-derived secretomes and exosomes; and (4) identify current challenges and translational barriers that limit clinical application. By integrating mechanistic insights with experimental and clinical findings, this review provides a comprehensive framework to inform future research, guide the design of standardized protocols, and support the development of MSC therapy as a viable strategy for enhancing fertility and productivity in cattle.

## SCOPE AND METHODS

This review was developed through a systematic search of the scientific literature focusing on the application of MSCs in treating reproductive disorders in cattle. Searches were conducted in major databases, including PubMed, ScienceDirect, Scopus, and Google Scholar, using keyword combinations such as *“mesenchymal stem cells,” “bovine,” “cattle,” “reproductive disorders,” “ovarian dysfunction,” “endometritis,” “infertility,”* and *“regenerative therapy.”* To capture recent advances, the search covered publications from 2010 to 2024.

Articles were included if they met the following criteria: (1) peer-reviewed and published in English; (2) focused on MSC therapy for female reproductive disorders in cattle or relevant animal models; and (3) comprised original research articles, reviews, or clinical trials. Exclusion criteria were (1) non-English publications, (2) studies not directly related to reproductive health or MSC applications, and (3) conference abstracts lacking sufficient data. Selected articles were further evaluated for scientific rigor and relevance to therapeutic mechanisms and clinical outcomes. Reference lists of included studies were also screened to identify additional relevant publications.

## MSC THERAPY ADVANCEMENTS FOR REPRODUCTIVE DISORDERS IN CATTLE

MSCs are multipotent cells capable of differentiating into diverse lineages, including bone, cartilage, and connective tissue [15]. Since their discovery in the late 20^th^ century, MSCs have become central to regenerative medicine research [16]. In veterinary applications, particularly bovine reproduction, MSCs have emerged as one of the most promising innovations [17]. Reproductive disorders such as ovarian dysfunction, estrous irregularities, and uterine abnormalities threaten productivity and animal welfare [18]. The application of MSCs offers an innovative approach to enhancing reproductive health, improving fertility outcomes, and strengthening livestock economics.

The first experimental applications of MSCs in reproductive disorders emerged in the early 2000s, coinciding with rapid advances in stem cell biology [19]. Early research focused on restoring ovarian function by repairing degenerative tissues and improving oocyte quality and yield [20]. Subsequent animal studies in cattle and ewes demonstrated that MSC therapy could reverse reproductive failure caused by ovarian or tract damage [21].

Recent biotechnological advances have further expanded the scope of MSC research. Between 2022 and 2024, studies emphasized tissue repair, restoration of ovarian microcirculation, and the creation of fertility-supportive microenvironments. Collectively, these findings establish MSCs as a transformative tool in advancing reproductive care in veterinary medicine [22, 23].

## REPRODUCTIVE DISORDERS IN CATTLE

Reproductive disorders significantly impair fertility in female cattle, leading to economic losses through reduced milk yield, lower conception rates, and decreased calf production. Such conditions often prolong calving intervals and increase perinatal mortality, further limiting herd productivity. Epidemiological reports suggest that 20%–30% of cattle within a herd may be affected by fertility-related issues at various stages of the reproductive cycle [24]. Delayed conception following calving or artificial insemination is a common problem, extending calving intervals and thereby reducing milk output and hindering calf growth [25]. A summary of the most frequently reported disorders is presented in Table 1 [9, 26–28].

**Table 1:** Common reproductive diseases in cattle.

S. No.	Diseases	Causes	Effect on reproduction	Reference
1.	Hormonal imbalance-induced infertility	Disruptions in estrogen, progesterone, and gonadotropin levels	Disrupt ovulation and pregnancy maintenance	[9]
2.	Cystic ovarian disease	Ovarian cyst formation that disrupts ovulation	Impairment of estrous cycles and infertility in cattle	[26]
3.	Endometritis	Uterine inflammation caused by bacterial infections	Impairs embryo implantation, leading to loss of pregnancy	[27]
4.	Age-related infertility	• Decline in oocyte number and quality • Disruptions in hormone production are essential for ovulation andpregnancy maintenance	• Lower conception rates and higher rates of pregnancy loss • Longer calving intervals and reduced productivity	[28]

## CHARACTERIZATION OF MSCS FOR REPRODUCTIVE THERAPY IN CATTLE

Proper characterization of MSCs is essential to ensure their suitability for therapeutic use in bovine reproductive disorders. Key attributes include their differentiation capacity, expression of specific surface markers, migratory ability, and immunomodulatory potential, which collectively determine their effectiveness in tissue repair and reproductive restoration [29].

A fundamental step in characterization is the evaluation of MSC adherence to culture surfaces under standard laboratory conditions [30]. The ability to attach to plastic culture dishes is a hallmark of MSC viability and quality, correlating with their survival, proliferation, and overall culture performance [31, 32]. This simple parameter serves as a baseline for determining the growth potential of MSC populations intended for therapy.

Flow cytometry provides further confirmation of MSC identity through the detection of cell surface markers. MSCs typically express Cluster of Differentiation 73 (CD73), CD90, and CD105 while lacking hematopoietic markers such as CD34 and CD45 [33, 34]. This phenotypic profile is critical for verifying purity, excluding contamination from non-mesenchymal cells, and ensuring consistency across therapeutic applications [35, 36].

The multipotent differentiation capacity of MSCs is another vital characteristic. They must be able to differentiate into multiple lineages, including osteoblasts, chondrocytes, and adipocytes [37, 38]. For reproductive applications, the ability to differentiate into granulosa and ovarian epithelial cells is particularly relevant, as it supports ovarian tissue regeneration and follicular development [39]. Induction under specialized culture media confirms this lineage-specific potential [40, 41].

Equally important is the homing ability of MSCs, which enables them to migrate toward injured or inflamed reproductive tissues. Guided by chemotactic signals, MSCs can localize to the uterus or ovaries and exert localized regenerative effects [42, 43]. This property has been validated through *in vivo* tracking methods, such as fluorescent or molecular labeling, which demonstrate MSC accumulation at sites of tissue injury and active participation in repair [44–46].

Ultimately, the immunomodulatory properties of MSCs are pivotal to their therapeutic efficacy. By secreting cytokines and growth factors, MSCs regulate immune responses, suppress excessive inflammation, and promote tissue repair [47, 48]. These effects are commonly assessed by analyzing cytokine profiles in culture and testing their ability to reduce inflammatory markers in experimental models [49].

Taken together, comprehensive characterization ensures that MSCs meet the required standards for use in bovine reproductive therapy. Their combined regenerative, homing, and immunomodulatory capabilities position MSCs as a compelling therapeutic option for restoring reproductive function in cattle [50].

## REGENERATIVE MSC MECHANISMS IN REPRODUCTIVE THERAPY

MSC therapy enhances bovine reproduction through a network of interconnected cellular and molecular mechanisms, including targeted migration, modulation of the reproductive microenvironment, and activation of regenerative signaling pathways [51].

The regenerative process begins with MSCs homing to injured sites in response to chemotactic cues such as stromal-derived factor-1α, which is secreted by inflamed or damaged tissues [52]. MSCs express receptors such as C-X-C chemokine receptor type 4 that recognize these signals, enabling directed migration and adhesion through integrins, cadherins, and selectins [44].

Upon reaching damaged tissues, MSCs actively remodel the local microenvironment by secreting bioactive factors [53]. They suppress excessive immune activity by downregulating T lymphocytes and pro-inflammatory M1 macrophages [54]. At the same time, they release anti-inflammatory cytokines such as interleukin (IL)-10 and transforming growth factor beta (TGF-β), which drive macrophage polarization toward the reparative M2 phenotype, while reducing levels of TNF-α and IL-6, thereby limiting chronic inflammation and tissue damage [55].

MSC therapy also promotes tissue regeneration through the secretion of growth factors, including vascular endothelial growth factor (VEGF), hepatocyte growth factor (HGF), and fibroblast growth factor (FGF) [56]. VEGF plays a pivotal role in angiogenesis, ensuring adequate oxygen and nutrient supply for regenerating tissue [57]. HGF and FGF stimulate the proliferation and migration of epithelial and stromal cells, facilitating repair of the endometrium and ovaries [58].

At the molecular level, MSCs activate several key signaling pathways essential for tissue regeneration. The phosphatidylinositol, 3-kinase/threonine-specific protein kinase B pathway enhances cell proliferation, inhibits apoptosis, and supports cell survival [59]. The Wnt/β-catenin pathway regulates differentiation and tissue restoration, particularly during endometrial repair [60]. In addition, MSCs interact with the TGF-β/Smad pathway, a regulator of fibrosis and extracellular matrix deposition. By suppressing this pathway, MSCs limit fibrotic tissue accumulation and preserve reproductive organ integrity [61, 62].

MSCs also protect reproductive tissues from oxidative stress. They enhance the activity of antioxidant enzymes such as superoxide dismutase and catalase, which reduce reactive oxygen species levels [63, 64]. Lower oxidative stress improves oocyte quality, supports folliculogenesis, and promotes ovarian recovery [65, 66].

Collectively, these synergistic processes, homing, immunomodulation, secretion of growth factors, regulation of signaling cascades, and antioxidative defense, work together to repair structural tissue damage, restore hormonal balance, and enhance fertility. By regenerating ovarian and endometrial tissues, improving oocyte quality, and supporting embryo implantation, MSC therapy provides a powerful approach to restoring reproductive function in cattle. Experimental studies have confirmed its efficacy in managing premature ovarian insufficiency, chronic endometritis, and polycystic ovarian syndrome through these complex and coordinated mechanisms [37, 67, 68].

## MSC THERAPY METHODS IN CATTLE

**Figure 1:**
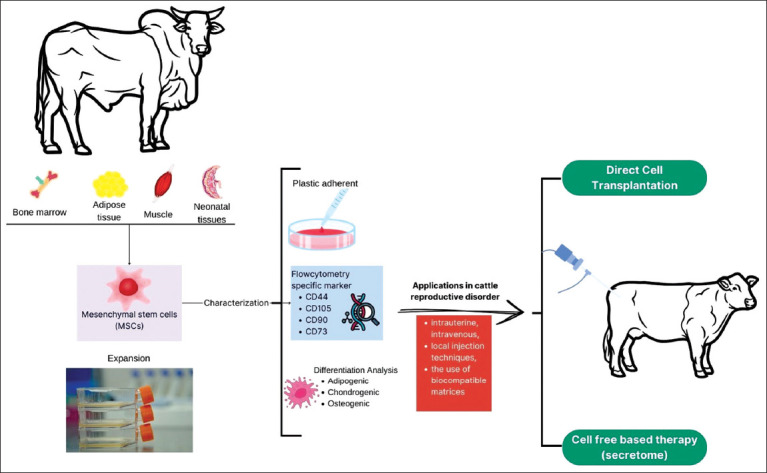
Schematic representation of mesenchymal stem cell (MSC) applications for treating cattle reproductive disorders. MSCs can be derived from various bovine tissues, including bone marrow, adipose tissue, muscle, and neonatal tissues. After isolation, the cells were expanded and characterized using flow cytometry based on their plastic adherence and expression of specific surface markers (CD44, CD105, CD90, and CD73). Multipotency is evaluated through adipogenic, chondrogenic, and osteogenic differentiation. Applications in reproductive therapy include intrauterine, intravenous, and local injection methods, which are often combined with biocompatible matrices. Two main therapeutic approaches are shown: direct cell transplantation and cell-free therapy using MSC-derived secretomes.

MSC-based therapy for bovine reproductive disorders is generally delivered through two main strategies: Direct MSC transplantation and secretome-based approaches using conditioned media or exosomes (Figure 1). The selection of the method depends on the clinical condition being treated and the desired therapeutic outcome [69]. Cellular MSC therapy is typically applied to directly repair damaged tissues through differentiation and local immunomodulation, while MSC-derived secretomes are increasingly used to exert strong paracrine effects that promote tissue regeneration without the risks of tumor formation or transplantation-related complications [70].

Intrauterine infusion is the preferred method for conditions such as endometritis and metritis [71]. Using specialized catheters under ultrasound or endoscopic guidance, MSCs are accurately delivered to the uterine lumen [72]. This facilitates direct interaction with damaged endometrial tissues and enhances regeneration through the secretion of paracrine factors such as VEGF and TGF-β, which stimulate angiogenesis and tissue repair [73]. However, this invasive approach carries risks of infection and requires specialized expertise [74].

Intravenous administration is commonly used for systemic reproductive disorders such as COD or chronic uterine infections [26]. This method is minimally invasive, technically straightforward, and adaptable to multiple conditions, as MSCs circulate through the bloodstream to reach reproductive tissues [75]. Nonetheless, sequestration of MSCs in non-target organs such as the lungs may reduce their delivery efficiency to reproductive sites, highlighting the need for optimized dosing and delivery protocols [76].

Intra-ovarian injection represents another effective approach, particularly for treating localized conditions such as follicular damage and ovarian degeneration [77]. Guided by transrectal ultrasound, MSCs are directly injected into ovarian follicles or damaged tissues [78]. This technique significantly enhances ovarian function and tissue integrity by stimulating follicular development and regeneration through MSC-secreted factors. However, it requires expert execution, and improper application may result in tissue trauma [79].

Beyond direct cell therapy, secretome-based approaches are gaining increasing attention. MSC-derived secretomes, including exosomes, microRNAs, and bioactive proteins, are harvested from conditioned media optimized for paracrine efficacy [80]. They can be administered intrauterine or intravenously, offering advantages such as lower immunogenic risk, greater ease of storage and transport, and reduced ethical concerns compared with live-cell therapies [81]. Exosomes, a major component of the secretome, regulate gene expression, modulate inflammation, and contribute to tissue regeneration [82]. However, paracrine effects are often transient, necessitating further studies to assess long-term therapeutic benefits.

Advances in MSC delivery methods continue to evolve, incorporating innovative strategies such as nanoparticle encapsulation, scaffold-based systems, and precision-guided delivery tools. These innovations aim to maximize regenerative efficacy, tailor therapy to individual physiological conditions, and minimize risks, ultimately improving reproductive health and productivity in cattle populations.

## EFFICACY OF MSC THERAPY FOR CATTLE REPRODUCTIVE DISORDERS

MSC therapy has demonstrated substantial efficacy in improving reproductive function, stimulating tissue regeneration, and reducing inflammation in cattle [83]. It is particularly effective against endometritis, metritis, COD, and infection-induced ovarian damage due to its combined immunomodulatory and regenerative effects [84].

Reported outcomes illustrate its therapeutic potential. For example, intrauterine MSC administration has improved pregnancy rates by up to 75% in cows with endometritis, while intra-ovarian injection has increased ovulation success by up to 80% in cases of ovarian cysts. Similarly, secretome-based therapies have achieved pregnancy rates of 65% in ovarian degeneration and 60% in ovarian dystrophy. These findings underscore the ability of MSC-based interventions to restore reproductive capacity through both cellular and paracrine mechanisms.

Over time, MSC therapy in cattle has progressed toward personalized approaches that focus on precise delivery, improved regenerative outcomes, and reduced complications [77, 78]. Delivery options now range from intrauterine and intravenous infusions to intra-ovarian injections, scaffold-supported applications, and secretome-based therapies [79], as summarized in Table 2, which outlines the application of stem cells for bovine reproductive disorders [85–89].

**Table 2:** Application of stem cells as a therapeutic approach for cattle reproductive disorders.

S. No	Study model	Reproductive disorder	Therapy type	Dosage/delivery method	Therapeutic outcome	Treatment pathway	Reference
1.	*In vivo* (dairy cattle)	Endometritis	Cells	1 × 10^6^ cells (intrauterine)	Reduced endometrial inflammation, increased expression of VEGF and TGF-β, endometrial regeneration, and improved pregnancy rate up to 75%	Immune modulation through IL-10, IL-6 inhibition, and angiogenesis stimulation	[85]
2.	*In vivo* (beef cattle)	Metritis	Secretome	500 µL exosome (intrauterine)	Uterine tissue recovery, increased sperm motility, and reduced post-insemination sperm DNA damage	Regulation of inflammation-related microRNA 21 and cell membrane repair	[86]
3.	*In vivo* (dairy cattle)	Endometriosis	Secretome	2 mL of exosome (intrauterine)	Reduced endometrial fibrotic lesion formation, improved endometrial epithelial structure, and estrous cycle recovery	Inflammation modulation through IL-4 and IL-13, inhibition of TGF-β	[86]
4.	*In vivo* (dairy cattle)	Ovarian Cyst	Cells	2 × 10^6^ cells (intraovarian)	Improved ovarian function, increased number of healthy follicles, and up to 80% ovulation success	Regulation of reproductive hormones (FSH and LH) and secretion of growth factor IGF-1	[87]
5.	*In vivo* (dairy cattle)	Ovarian degeneration	Secretome	1 mL secretome (intravenous)	Increased follicular activity, reduced ovarian fibrosis, and improved pregnancy success after artificial insemination (up to 65%)	Reduction of oxidative stress through superoxide dismutase and GPx and enhancement of local angiogenesis	[88]
6.	*In vivo* (dairy cattle)	Ovarian dystrophy	Secretome	1 mL secretome (intraovarian)	Increased production of estradiol hormone, follicular activity recovery, and improved pregnancy rate up to 60% after artificial insemination	Regulation of reproductive hormone pathways through follicle-stimulating hormone and IGF-1	[89]

FSH = Follicle-stimulating hormone, LH = Luteinizing hormone, IGF-1 = Insulin-like growth factor 1, GPx = Glutathione peroxidase, IL = Interleukin, TGF-β = Transforming growth factor-beta

Although intrauterine infusion remains the preferred method for uterine disorders such as endometritis and metritis [27], it requires invasive techniques and trained personnel, which limit broader clinical adoption [28]. Intravenous administration provides a minimally invasive alternative for systemic conditions [85, 86], though its efficiency is restricted by cell sequestration in non-reproductive organs. In contrast, intra-ovarian delivery is highly effective for localized damage, directly enhancing ovarian quality and follicular activity [87–89].

Secretome-based therapies represent an emerging frontier, offering a safe, cell-free alternative with lower immunogenicity and easier application compared with live MSCs [80, 81]. While highly promising, further research is needed to extend their short-lived effects and confirm their long-term reproductive benefits [82].

Collectively, these findings demonstrate that MSC therapy, whether through cellular transplantation or secretome delivery, provides a transformative approach to restoring fertility and advancing reproductive biotechnology in cattle.

## CURRENT MSC THERAPY CHALLENGES FOR REPRODUCTIVE DISORDERS IN CATTLE

Despite its promising potential, MSC therapy in cattle faces several challenges that must be addressed before it can be widely adopted in clinical practice [90]. One of the primary limitations is the variability in therapeutic response, which is influenced by factors such as breed, age, and overall health status [91]. For instance, older animals or those suffering from chronic infections tend to respond less favorably compared to younger and healthier cattle. This variability complicates the establishment of standardized dosages and administration protocols across diverse cattle populations [90].

Another significant barrier is the difficulty in ensuring precise delivery of MSCs to reproductive tissues. Although intrauterine, intravenous, and intramuscular routes have been tested, their relative effectiveness is inconsistent [92, 93]. Systemic administration, in particular, poses the risk of MSC entrapment in non-target organs, which reduces the proportion of cells reaching the intended reproductive sites and diminishes therapeutic efficacy [94]. Optimizing delivery techniques remains a critical need.

In addition to technical challenges, regulatory and biosafety concerns present major obstacles. MSC applications in livestock are tightly regulated under national and international frameworks, and the complexity of these regulations can delay clinical approval [95, 96]. Robust safety validation, including comprehensive preclinical and clinical trials, is required to confirm the efficacy and biosafety of MSC therapies [97]. Furthermore, potential risks, including tumorigenesis, infection, and adverse immunogenic reactions, must be carefully assessed and minimized before clinical adoption [98].

Finally, the requirement for long-term monitoring adds another layer of complexity. Surveillance is essential to detect delayed systemic effects or latent alterations in reproductive tissues following MSC administration, ensuring the sustained safety and effectiveness of therapy [99].

Taken together, these challenges highlight the urgent need for standardized protocols, improved delivery systems, and harmonized regulatory frameworks to enable the safe and effective translation of MSC therapy from experimental studies to routine veterinary practice.

## CONCLUSION

This review highlights the growing potential of MSCs as a regenerative tool for managing reproductive disorders in cattle. Evidence from preclinical and clinical studies shows that MSCs exert therapeutic benefits through multiple mechanisms, including homing to injured tissues, secretion of bioactive factors, modulation of immune responses, stimulation of angiogenesis, antifibrotic activity, and reduction of oxidative stress. Together, these mechanisms contribute to ovarian and endometrial tissue repair, improvement of oocyte quality, enhanced embryo development, and restoration of fertility.

MSC-based interventions, delivered through intrauterine infusion, intravenous administration, or intra-ovarian injection, have demonstrated measurable improvements in pregnancy rates, ovulation success, and overall reproductive efficiency in cattle. Cell-free approaches using secretomes and exosomes provide additional practical options, offering advantages in safety, storage, and immunological compatibility. These therapies hold the potential to improve herd fertility, reduce reliance on antibiotics, and enhance livestock productivity, thereby supporting animal health and agricultural sustainability.

The strength of MSC therapy lies in its dual regenerative and immunomodulatory functions. Unlike conventional treatments that primarily manage symptoms, MSCs address underlying pathology by restoring tissue integrity and hormonal balance. Their adaptability across delivery methods and multipotent nature further reinforce their therapeutic value in veterinary reproductive medicine.

Nevertheless, challenges remain that limit clinical translation. Variability in treatment response across breeds, ages, and health conditions complicates the standardization of treatment. Technical issues in achieving precise delivery, possible risks of immunogenicity or tumorigenesis, high production costs, and stringent regulatory frameworks continue to hinder broader clinical adoption. Moreover, much of the available evidence remains preclinical, with only limited large-scale clinical validation in cattle.

Future research should focus on optimizing delivery strategies, standardizing dosing protocols, and integrating advanced technologies such as biomaterials and nanocarriers to improve targeting efficiency. Further exploration of engineered secretomes and exosomes will be crucial to establish their long-term therapeutic efficacy and scalability. Large-scale, controlled clinical trials are urgently needed to confirm safety, economic feasibility, and regulatory compliance.

Overall, MSC therapy holds transformative potential for reproductive biotechnology in cattle, provided that safety, efficacy, and regulatory frameworks are addressed in parallel. Combining regenerative medicine with veterinary practice offers a sustainable and innovative alternative to conventional therapies, with the potential to revolutionize fertility management in livestock. To achieve this promise, future work must bridge current experimental findings with safe, standardized, and cost-effective clinical applications.

## AUTHORS’ CONTRIBUTIONS

TAP, SSu, and ZM: Data curation, formal analysis, development of the methodology, and drafted and revised the manuscript. SSu, HH, TPP, and SSa: Conceptualization, project management, and supervision. TK, SAA, and DAK: Formal analysis and data curation. PIS, MFH, and NA: Visualization and critical review of the manuscript. All authors have read and approved the final version of the manuscript.
